# Watchman Device Procedure Complicated by Rare Perclose ProGlide Embolization: Case Report and Literature Review

**DOI:** 10.7759/cureus.21173

**Published:** 2022-01-12

**Authors:** Rafail Beshai

**Affiliations:** 1 Internal Medicine, Jefferson Health New Jersey, Stratford, USA

**Keywords:** vascular closure device, embolization, proglide, perclose, watchman, watchman device

## Abstract

Atrial fibrillation (AF) is the most common cardiac arrhythmia with significant morbidity and mortality. In this case, we present a 69-year-old man with a past medical history of atrial fibrillation on warfarin who came to the hospital for placement of the Watchman device (Boston Scientific Corporation, Marlborough, Massachusetts, United States). His procedure was complicated by Perclose ProGlide™ Suture-Mediated Closure system (Abbott Laboratories Inc., Chicago, Illinois, United States) embolism. The vascular surgery department was consulted STAT in the electrophysiology lab and was unable to fully visualize the vessel. The patient was then brought emergently to a hybrid operating room where venotomy was made. He tolerated the procedure well and was eventually discharged home.

## Introduction

Atrial fibrillation (AF) is the most common cardiac arrhythmia [1]. It is the leading cause of cardiac stroke with significant morbidity and mortality [2]. It is well established that patients with AF have a five-fold increased risk of stroke compared to those without, and that anticoagulation reduces the risk of stroke by approximately two-thirds [3]. Oral anticoagulants (OACs) are used in the prevention of stroke in patients with AF [4]. However, a substantial number of patients have relative or absolute contraindications to OACs due to concerns of major bleeding risk and other adverse effects while using the OAC therapy [4]. The left atrial appendage (LAA) is the main source of thrombus formation in AF patients. The Watchman device (Boston Scientific Corporation, Marlborough, Massachusetts, United States) works by closing the LAA so the procedure serves as a promising, safe, and effective alternative to OAC, overcoming disadvantages including the risk of major bleeding. Vascular closure devices (VCDs) like Perclose ProGlide™ Suture-Mediated Closure System (Abbott Laboratories Inc., Chicago, Illinois, United States) have largely replaced the use of mechanical compression in managing femoral access after Watchman device procedures [5]. The device has demonstrated excellent safety. Breaking of the device intra-operatively is a very rare complication that providers should be aware of.

## Case presentation

A 69-year-old male with a past medical history of persistent AF on warfarin presented to the hospital for cardiac electrophysiology (EP) evaluation for placement of the Watchman device. He had frequent and regular falls and problems with labile international normalized ratio (INR) due to noncompliance with his INR follow-up. The patient was brought to the electrophysiology laboratory in the fasting state. He was prepped and draped in the usual sterile fashion after a transesophageal echocardiogram (TEE) revealed no evidence of left or right atrial appendage clot. Following the induction of general anesthesia, access was obtained to the right femoral vein with good blood flow, and preclose technique was planned by placing the Perclose ProGlide suture at the beginning of the procedure; knot advancement was placed on hold until the procedure is complete. During Perclose deployment, the device sutures did not come back out and the footplate was stuck in the open position. Attempts at rotation, advancement, opening, and closing the footplate were all unsuccessful at mobilizing the device. Ultrasound showed the footplate as opposed to the vessel wall without movement. 

The vascular surgery department was called STAT to the room and performed a limited cutdown in the EP lab confirming the Perclose system in the right common femoral vein but was unable to fully visualize the vessel. The patient was brought emergently from the EP lab to a hybrid operating room. There was a closure device protruding from the right femoral vein. An extended dissection was carried down through subcutaneous tissue. Proximal and distal control were obtained of the common femoral, superficial femoral, profunda femoris, and saphenous veins. Venotomy was made and the closure device was removed. It appeared that the closure device had broken in half, with the midportion getting hooked on the vein, and that is why could not be removed and the other half embolized about 5 cm more proximal. Next, the saphenous vein and the common femoral vein were thrombectomized. The clot was sent to pathology and the device was sent to the company. The patient was taken to the ICU following his surgery where he was placed under close clinical monitoring and serial vascular exams. He was eventually discharged home. He was advised to keep his INR appointment, and he showed understanding and agreement.

## Discussion

VCDs are widely used in femoral access cardiovascular procedures for early ambulation, improving patient comfort, and potential morbidity reduction [6]. The Perclose ProGlide Suture-Mediated Closure System is one of the commonly used VCDs, which has evolved since 1994 [7]. Perclose ProGlide is considered safe overall and publications describing its side effects including its breakage and embolization are very limited [8]. To our knowledge, only two case reports were found on PubMed and Google Scholar describing breakage and embolization of Perclose ProGlide VCD (Table 1). We used the terms "Perclose ProGlide," "vascular closure device," with "embolization" and "breakage." We believe that this is the first case to describe Perclose ProGlide breakage and embolization in the setting of the Watchman device placement procedure.

**Table 1:** Previous cases of Perclose ProGlide Embolization PPSMC: Perclose ProGlide™ Suture-Mediated Closure

Name of the author	Year	Age/sex of patient	Type of Procedure in which PPSMC was used
Giniyani et al. [5]	2020	87/M	Elective endovascular repair of aortic aneurysm
Kang et al. [9]	2015	76/M	Right and left heart catheterization for heart failure

Embolization of intravascular foreign bodies poses many risks for our patients including the development of perforation, septic complications, thrombosis, and obstruction in blood flow [10]. In the previous two cases of Perclose ProGlide embolizations, a snare retrieval approach was used to manage this complication [5,9]. In our case, the patient needed to go to the operating room emergently. Even though Perclose ProGlide is an extremely safe product, clinicians should be aware of the rare complication of the procedure. Although further studies are warranted, we propose here that STAT vascular surgery consults in these instances are needed and could be lifesaving.

## Conclusions

Although Perclose ProGlide embolization is very rare, it is potentially fatal. Consequently, clinicians should be aware of this complication of the Watchman device implant procedure.
